# Adsorption of lithium ions from aqueous solution by magnetic aluminum-based adsorbents

**DOI:** 10.1371/journal.pone.0295269

**Published:** 2023-12-01

**Authors:** Yaru Qin, Tingfei Yang, Chenglong Shi, Bing Liu

**Affiliations:** 1 School of Chemistry and Chemical Engineering, Qinghai Minzu University, Xining, Qinghai, China; 2 Department of Chemistry, School of Science, Tianjin University, Tianjin, China; 3 College of Chemical Engineering, North China University of Science and Technology, Tangshan, Hebei, China; Saveetha Institute of Medical and Technical Sciences: Saveetha University, INDIA

## Abstract

Magnetic aluminum-based adsorbents (MLDHs) were prepared with a coprecipitation method and used to separate lithium ions from the aqueous solutions. In static adsorption experiment, the adsorption capacity of MLDHs for lithium ions reached 8.22 mg/g. In a mixed solution of various metal ions, the adsorbents exhibited higher selectivity for lithium ions. Kinetic studies indicated that the adsorption process conformed to a pseudo-second-order model. The experimental data were fitted with nonlinear regression using commonly used adsorption isotherms. It was found that the adsorption isotherm process could be described by the Langmuir model. In addition, the thermodynamic parameters revealed that the adsorption of lithium was a spontaneous endothermic process.

## 1. Introduction

As an important strategic resource to reduce carbon emissions, lithium is known as "the metal that drives the world forward". Lithium and its compounds play an important role in many fields, such as new energy vehicles, glass, metallurgy, batteries, etc [1–6]. Lithium resources were mainly found in solid ores and salt lakes. The main problems of lithium extraction from ores were high energy consumption, high cost, and difficult tailings treatment. Since about 75% of lithium on land is stored in brine, lithium recovery from aqueous solutions has a significant advantage [7]. The main methods for lithium extraction from solution include evaporation and precipitation, solvent extraction, adsorption, and membrane separation [8–13]. Among them, the adsorption method has the advantages of high selectivity and simple operation, which is a promising method [14].

At present, the lithium adsorbents used to extract lithium ions mainly included titanium-based, manganese-based, and aluminum-based adsorbents [15, 16]. Titanium-based and manganese-based adsorbents exhibited excellent lithium adsorption properties, but under acidic conditions, the adsorbents were easily dissolved and lost and the adsorbent structure collapses, which limited their industrial applications [17–21]. Aluminum-based sorbents (LDHs) are the most widely studied inorganic adsorbents due to their stable cycle performance and easy preparation [22, 23]. Zhong et al [24, 25] prepared Li/Al-LDHs with a dimensional hexagonal plane, and investigated the effect of excessive lithium deintercalation on their adsorption performance. Experiments showed that the adsorption capacity of Li^+^ was 7.27 mg/g. It was confirmed that Li/Al-LDHs gradually transformed into gibbsite with excessive deintercalation, and the collapse intensity of the Li/Al-LDHs layered structure was positively correlated to excessive deintercalation intensity. When Fe(III) was doped into Li/Al-LDHs, it would enhance the stability of Li/Al-LDHs and reduce its solubility in nanocomposites [26]. The Li_x_Al_2_-LDH adsorbents were prepared by Lee et al [27]. The Li_x_Al_2_-LDH@SiO_2_ nanostructure showed enhanced Li^+^ adsorption performance and high Li^+^ selectivity in mixed water resources of Mg^2+^, Na^+^, and K^+^ cations. They further proposed a new lithium deintercalation method [28]. The results indicated that lithium ions undergo topological reactions between amorphous Li-Al-O-OH and LiAl_2_-LDH. A series of LDH adsorbents were synthesized and applied to extract lithium from geothermal brine. A Lithium recovery efficiency of approximately 91% was achieved [29].

In practical industrial applications, the synthesis of granular sorbents using organic binders was essential considering the difficulties in the separation and recovery of powdered aluminum-based adsorbents. However, the granulation process was tedious, the cost increased, and especially the adsorption performance decreased dramatically [30]. Kotsupalo et al. [31] prepared the granular LDHs using chlorinated polyvinyl chloride resin as a binder. The granular LDHs could selectively adsorb lithium ions from salt lake brine, however, the adsorption capacity of lithium ions decreased to 1.24 mg/g. Zhong et al. [32] developed a hybrid binder to granulate powdered LDHs into granular LDHs with applicable mechanical strength. The adsorption capacity of granular LDHs for lithium ions reached 4.92 mg/g at 303K. In order to avoid the decrease of LDHs adsorption capacity caused by the inefficient granulation process, a new strategy was designed by doping superparamagnetic nanoparticles (Fe_3_O_4_@SiO_2_) in the LDHs to achieve rapid recovery of adsorbents with the help of a magnetic field.

In this paper, the magnetic aluminum-based adsorbents (MLDHs) were prepared by homogeneously doping silicon dioxide coated ferric tetroxide nanoparticles (Fe_3_O_4_@SiO_2_) into the aluminum-based adsorbents. The adsorption performance of the MLDHs for Li^+^ in solution was investigated. The selectivity of the adsorbents to lithium ions in a mixed solution was verified. The mass transfer mechanism of the adsorption reaction was determined by kinetic models. In addition, the equilibrium data were analyzed using an isothermal model, resulting in thermodynamic parameters.

## 2. Experiments and methods

### 2.1 Materials and instruments

The chemicals, aluminum chloride hexahydrate (AR), potassium chloride (AR), monodispersed magnetite microspheres (AR), sodium chloride (AR), lithium hydroxide monohydrate (AR), magnesium chloride hexahydrate (AR), tetraethyl orthosilicate (AR), and lithium chloride (AR), were purchased from Aladdin Industrial Corporation (China). The temperature of the adsorption process was controlled by a water bath oscillator. The structure of MLDHs were characterized by an X-ray diffractometer (Rigaku Ultima IV) and scanning electron microscope (Zeiss Sigma 500). The concentrations of metal ions were determined with an inductively coupled plasma atomic emission spectrometer (Thermo Scientific ICAP-Pro).

### 2.2 Synthesis of MLDHs

The Fe_3_O_4_ particles (0.15 g) were dispersed into a mixed solvent of ethanol and water with a volume ratio of 4:1, and then ammonia (7 mL, 20% wt) was added to the solvent. Fe_3_O_4_@SiO_2_ nanoparticles were prepared by adding ethyl silicate (TEOS) (4 mL) to the solution at room temperature and stirring for 10 h. Then, the Fe_3_O_4_@SiO_2_ nanoparticles can be separated from the solution. The obtained nanoparticles were washed three times with ethanol and water, and dried at 333K for 10 h.

The aluminum trichloride hexahydrate (3.13 g) and lithium hydroxide monohydrate (0.27 g) were dissolved in deionized water to form a mixed solution. The 2.00 mol/L NaOH solution was added dropwise to the mixed solution, and the pH at the end of the reaction was controlled to be 6.5. The above mixed solution was stirred and reacted for 2 h to obtain a suspension. The suspension was centrifuged, and the solid obtained was washed thoroughly 5 times with deionized water, dried at 60°C to obtain LDHs.

**Fig 1:**
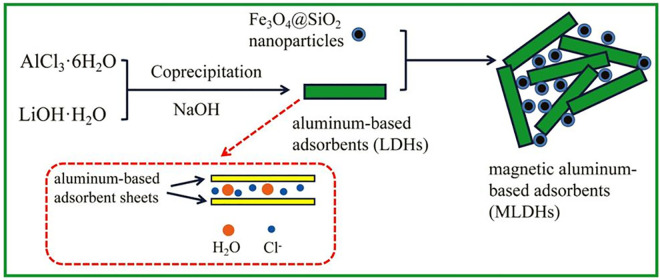
Schematic diagram of the preparation of MLDHs.

The obtained Fe_3_O_4_@SiO_2_ nanoparticles (0.2 g) were added into deionized water and dispersed by ultrasonic to form a suspension. Then, the LDHs (0.8 g) was added to the suspension, and the suspension was continued to disperse 30 min by ultrasonic. After filtration and drying, the final product MLDHs was obtained. Finally, the obtained solids were filtered and dried at 60°C overnight to gain the MLDHs. The synthesis strategy of MLDHs was described by the scheme in Fig 1.

### 2.3 Adsorption experiment

The adsorption behavior of the MLDHs for lithium ion in solution was studied by the static adsorption method. All adsorption studies were performed at 293 K, except for the experiments used to determine thermodynamic parameters at variable temperatures. A certain amount of MLDHs were weighed and placed in an aqueous solution containing lithium, which was shaken on an oscillator for a certain time and then separated from the liquid by an applied magnetic field. The solution before and after adsorption was taken out and diluted to measure the ion concentrations. The adsorption capacity q_e_ (mg/g), partition coefficient K_d_ (L/g) of lithium ions, and selectivity factor α were obtained from the following formulas:

$$q_{e}=\frac{(C_{0}-C_{e})\cdot V}{m}$$ (1)

$$K_{d}=\frac{\left(C_{0}-C_{e}\right)}{C_{e}}∙\frac{V}{m}$$ (2)

$$\alpha_{Me}^{Li}=\frac{K_{d,Li}}{K_{d,Me}}$$ (3)

where *C*_*0*_ (mg/L) and *C*_*e*_ (mg/L) were the initial and residual lithium concentrations, respectively. The m (g) was the mass of the MLDHs and V (L) was the volume of the aqueous solution containing lithium. The subscript Me of α represents Na^+^, K^+^, Mg^2+^, and Ca^2+^.

## 3. Results and discussion

### 3.1 Characterization of MLDHs

**Fig 2:**
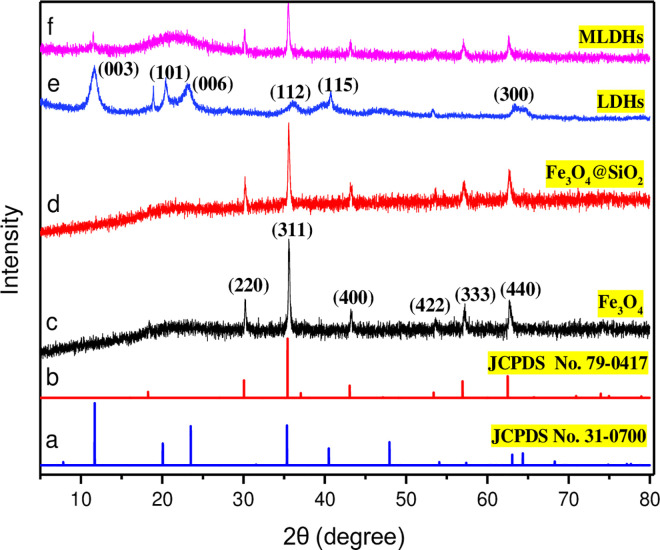
XRD patterns of (c) Fe_3_O_4_, (d) Fe_3_O_4_@SiO_2_, (e) LDHs and (f) MLDHs. (a) JCPDS card of LDHs; (b) JCPDS card of Fe_3_O_4_.

The X-ray diffraction spectrum and characteristic peaks of Fe_3_O_4_, Fe_3_O_4_@SiO_2_, LDHs and MLDHs were shown in Fig 2. As shown in Fig 2C and 2D, apparent peaks were observed at 30.35°, 35.54°, 43.29°, 53.61°, 57.14° and 62.68° degrees which correspond to (220), (311), (400), (422), (333) and (440) face centred cubic crystal orientation, respectively [33]. The spectra were in close agreement with JCPDS card No.79-0417. Since SiO_2_ was in amorph structure, no SiO_2_-related peaks were observed in the XRD pattern of Fe_3_O_4_@SiO_2_ particles [34]. Representative diffraction peaks at 2θ = 11.51°, 20.44°, 23.14°, 36.09°, 40.66° and 63.18° can be clearly identified (Fig 2E). Compared with the XRD spectrum standard card (JCPDS No. 31–0700), it was known that MLDHs accorded with the chemical formula LiCl·2Al(OH)_3_·xH_2_O, and its XRD reflections indexed to the crystal plane of (003), (101), (006), (112), (115) and (300). However, most of these representative diffraction peaks were not observed in the diffraction patterns of MLDHs. Fig 2F displayed a new broad hump around 21.11°. The characteristic diffraction peak of MLDHs at 11.51° was attributed to the LDHs crystal phase, and the intensity was weak. The reason may be that Fe_3_O_4_@SiO_2_ particles were dispersed in the interlayer of LDHs, which reduced the crystallinity of LDHs, resulting in its diffraction peaks not appearing in the diffraction pattern of MLDHs.

**Fig 3:**
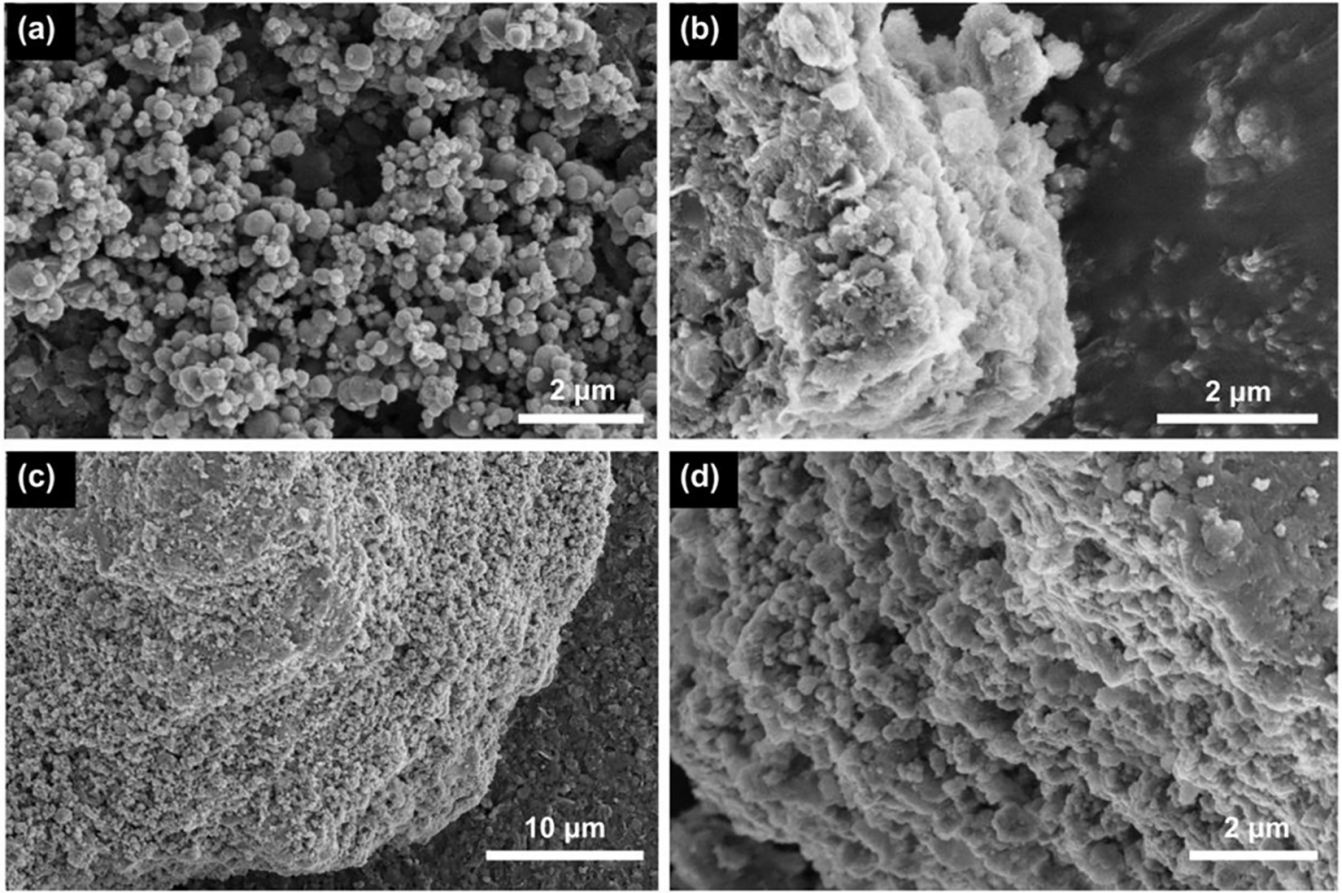
The SEM images of Fe_3_O_4_@SiO_2_, LDHs and MLDHs. (a) Fe_3_O_4_@SiO_2_; (b) LDHs; (c) and (d) MLDHs.

**Fig 4:**
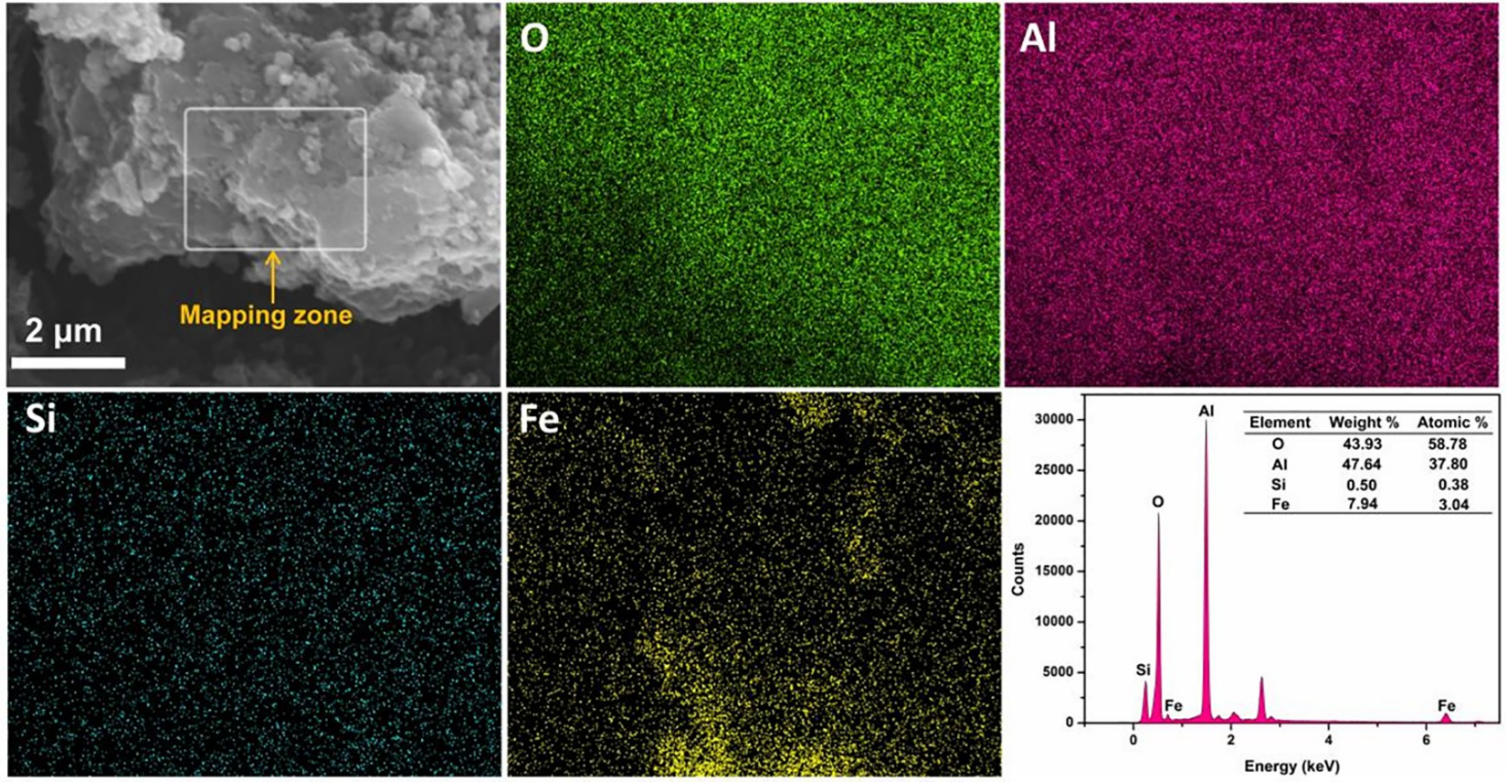
The EDS analysis of MLDHs.

The SEM analysis of Fe_3_O_4_@SiO_2_, LDHs, and MLDHs were illustrated in Fig 3. As shown in Fig 3A, the Fe_3_O_4_@SiO_2_ particles had a sphere-like morphology and some of the particles were found to be in agglomerated form. The SEM image in Fig 3B demonstrated a legible layered structure of LDHs. As displayed in Fig 3C, numerous Fe_3_O_4_@SiO_2_ particles can be observed dispersed on the surface of the LDHs. Fig 3D showed the morphology at the cross-section of the layered structure of LDHs. It can be observed from Fig 3D that Fe_3_O_4_@SiO_2_ particles can be dispersed in the interlayer of LDHs and form many small pores in the adsorbent structure. The energy dispersive X-ray spectroscopy (EDS) of the MLDHs particles were depicted in Fig 4. The EDS spectrum demonstrated that Al, O, Fe and Si elements were presented in the structure of the MLDHs particles. The results of SEM and EDS analysis proved that Fe_3_O_4_@SiO_2_ particles were successfully combined with LDHs to form MLDHs.

### 3.2. Adsorption studies

#### 3.2.1 Effect of pH on lithium adsorption

**Fig 5:**
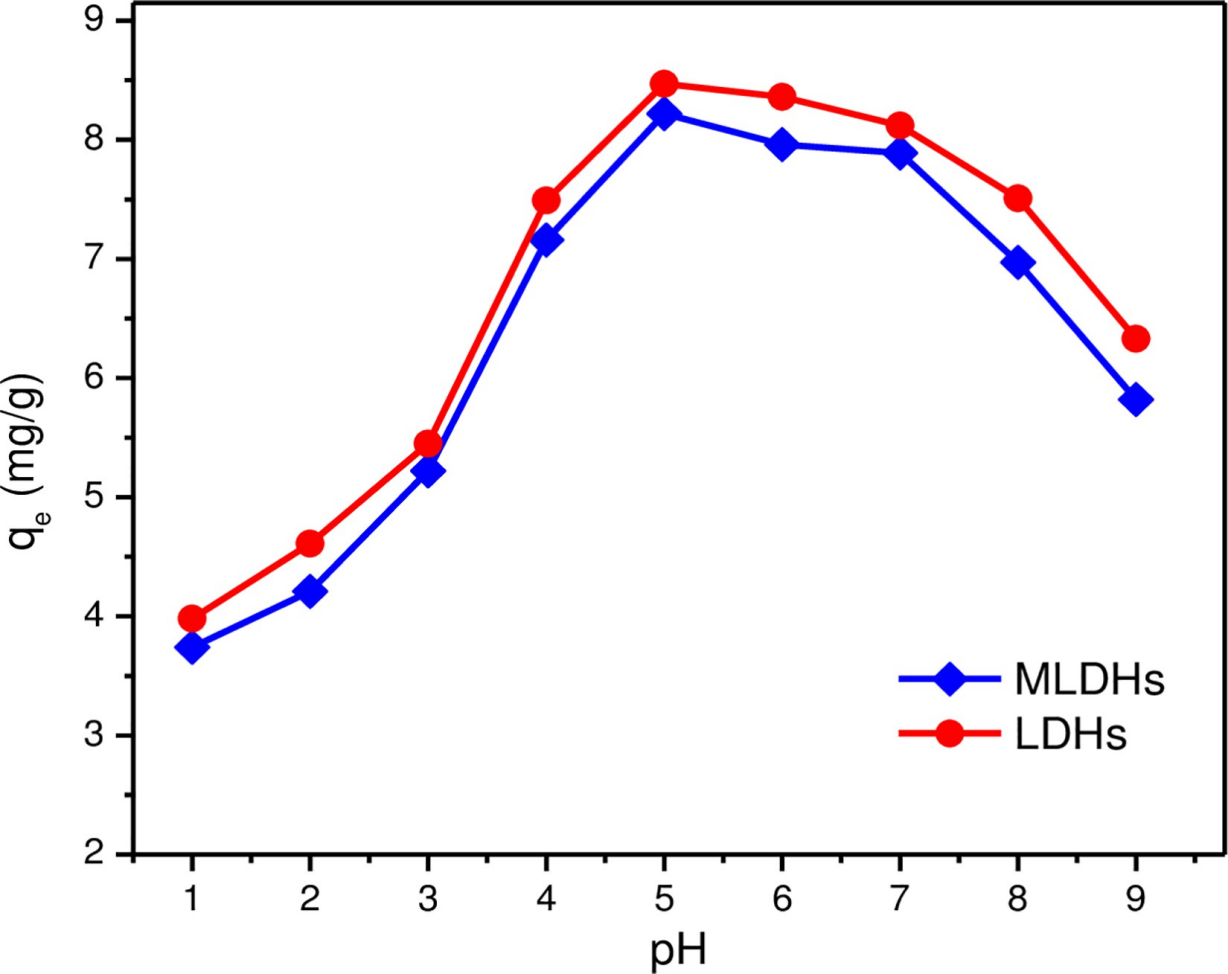
Effect of pH on adsorption capacity.

Since aluminum hydroxide was a typical amphiphilic hydroxide, the solution acidity had a great influence on the stability of MLDHs. If the acidity of the solution was high, the adsorbent may decompose. However, the adsorbent will be converted to meta-aluminate when the acidity was low. The adsorption capacities of MLDHs were investigated by adding 0.2 g MLDHs into 15 mL pure lithium chloride solution.The initial Li^+^ concentration was 0.40 g/L and the Cl^-^ concentration was 2.04 g/L. Fig 5 showed the variation trend of adsorption capacity. When the acidity of the solution weakened, the adsorption capacity of MLDHs for lithium ions increased at first and then decreased gradually. At a pH of 5, the adsorption capacity of lithium ions reached a maximum. The structure of the adsorbent will be destroyed if the acidity of the aqueous solution is too high. In addition, there is competitive adsorption between H^+^ and Li^+^, which will lead to a decrease in adsorption capacity. The adsorption performance of LDHs was also investigated. It can be seen from Fig 5 that the adsorption process of MLDHs has a similar trend to that of LDHs. Under the same experimental conditions, the adsorption capacity of LDHs was slightly higher than that of MLDHs. In addition, it was found that pure Fe_3_O_4_@SiO_2_ particles have no adsorption capacity for Li^+^, which indicated that LDHs were effective adsorption components in MLDHs. However, it was difficult to separate and recover powdered LDHs in application. If the powdered LDHs were granulated and formed by adding organic binder, the adsorption performance will decrease rapidly. The MLDHs had good adsorption capacity and can be recovered quickly under an external magnetic field. Considering the stability and adsorption capacity of the adsorbents, the pH value of the subsequent experimental solution was 5.

#### 3.2.2 Effect of lithium ion equilibrium concentration on adsorption capacity

**Fig 6:**
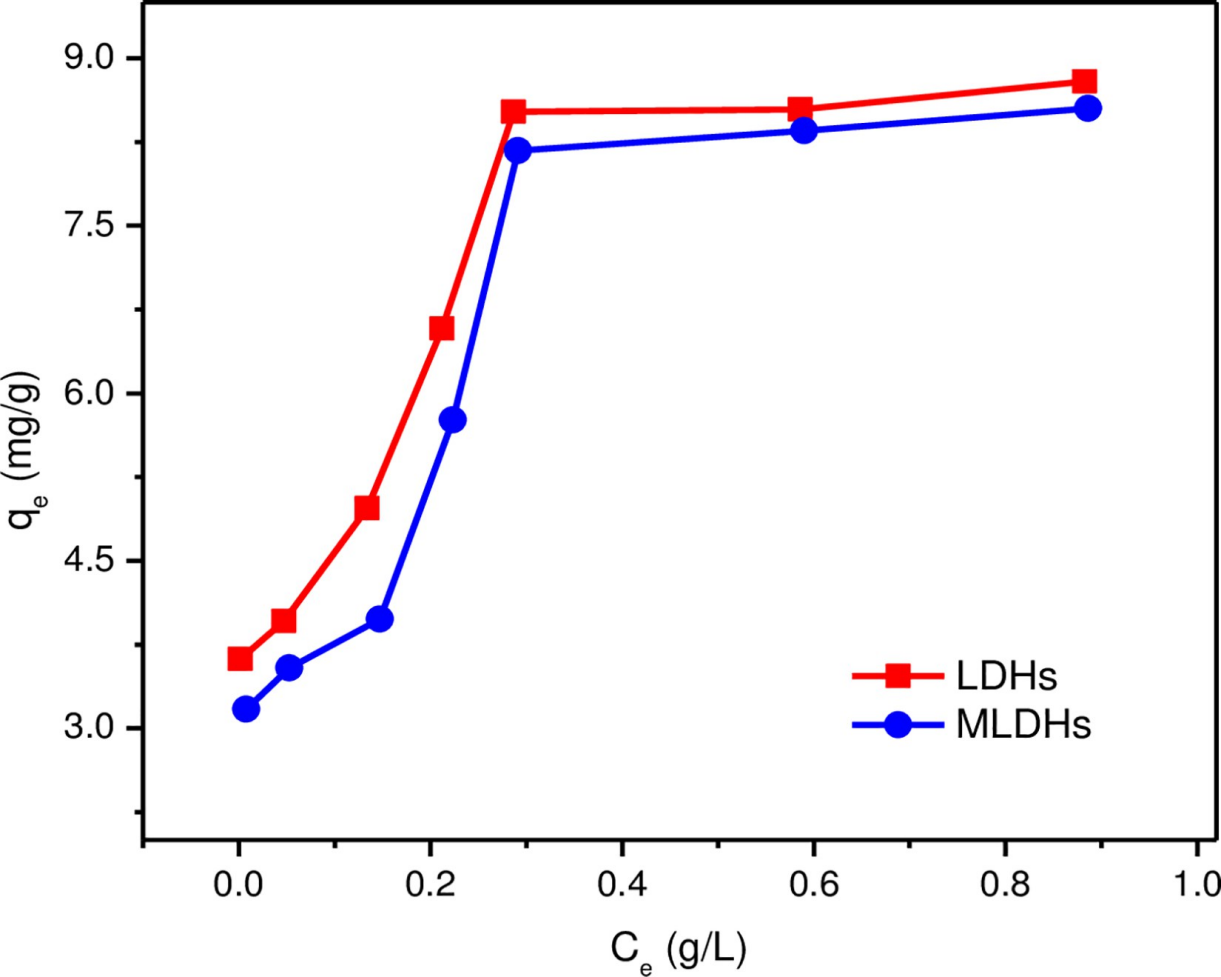
Effect of initial lithium ion concentration on adsorption capacity.

The Fig 6 showed the effect of equilibrium concentration on lithium adsorption capacity. The initial Li^+^ concentration was varied from 0.05 g/L to 1.00 g/L, and the Cl^-^ concentration was varied from 0.26 g/L to 5.11 g/L. It can be seen from the adsorption capacity that the high lithium ions concentration exerted a positive influence on adsorption. At the fixed solid-liquid ratio, the adsorption capacity increased with the increase of Li^+^ concentrations. This was due to the fact that the lithium ion concentration difference was the main driving force for adsorption. When the concentration of lithium ions was high, it was beneficial for lithium ions to quickly occupy the adsorption sites, thus showing a high adsorption capacity. Therefore, in the actual adsorption process, the lithium-containing solution needed to be properly concentrated to increase the adsorption capacity.

### 3.3 Adsorption mechanism

Previous studies have shown that aluminum-based adsorbents have a two-dimensional layered structure, and the layers are connected by forces such as electrostatic interactions and hydrogen bonds [35, 36]. In the process of preparing aluminum-based adsorbents, the adsorbents are usually washed with deionized water to remove a fraction of Li^+^ in the structure and create vacancies in the adsorbent structure. These vacancies serve as the adsorption sites for lithium ions, and their size matches the lithium ions best, thus exhibiting a high selectivity for Li^+^ [37–39]. In addition, the hydration free energy of lithium ions was much lower than that of magnesium ions, and the adsorbents were more likely to combine with lithium ions, thereby achieving the purpose of separating lithium and magnesium [1]. The mechanism of adsorption and desorption of lithium by aluminum-based adsorbents could be described by the following Eq (4) [40].


$$xLiCl+(1-x)LiCl\cdot mAl(OH{)}_{3}+(n+1)H_{2}O⇌LiCl∙mAl(OH{)}_{3}∙nH_{2}O+H_{2}O$$ (4)

**Fig 7:**
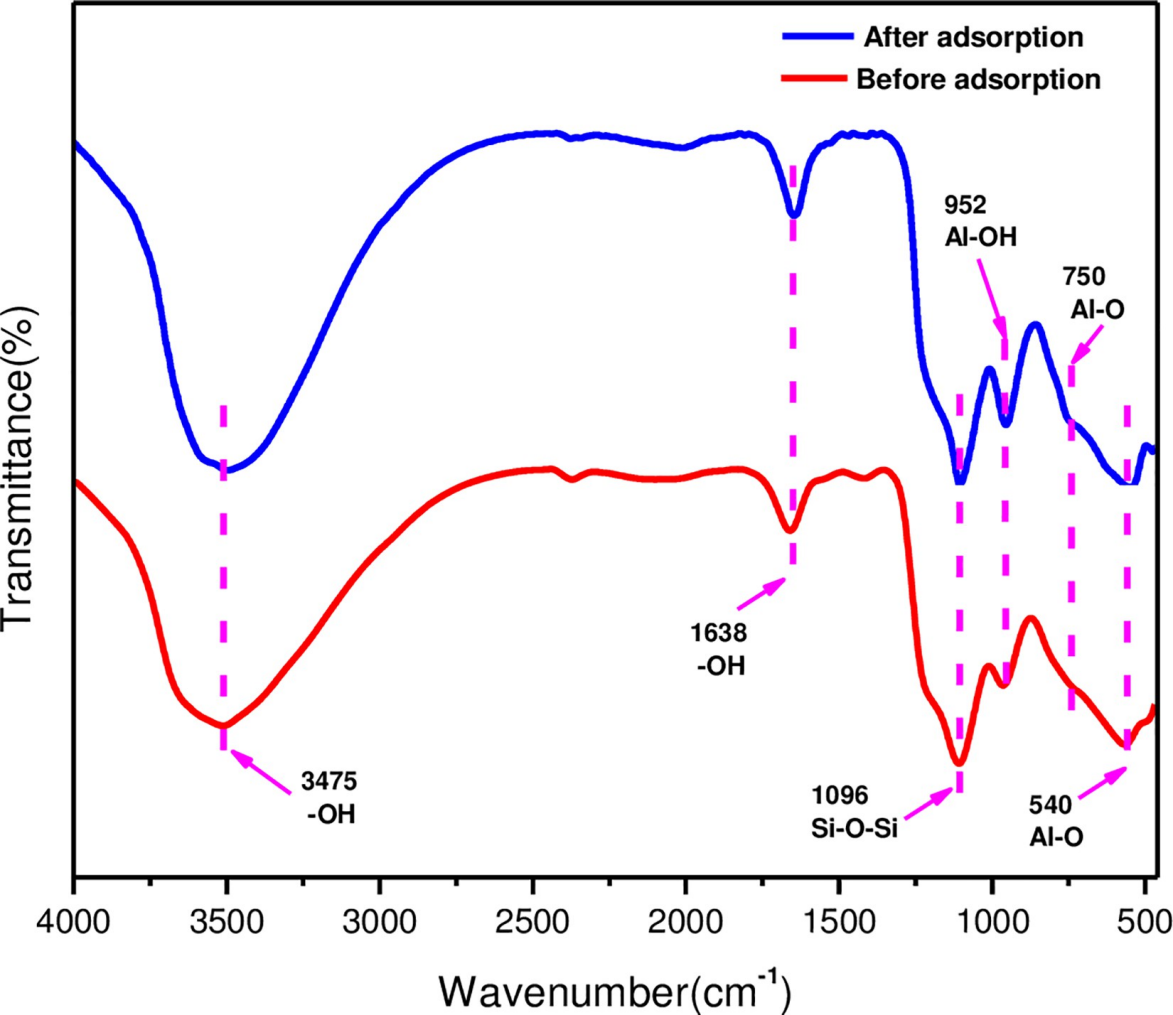
FTIR spectra of MLDHs.

The FTIR spectra in Fig 7 characterized the change of the absorption peak of the functional group of the MLDHs before and after adsorption. The broad absorption peak at 3475 cm^-1^ could be considered as the stretching vibration of the O-H bond. The deformation vibration absorption peak of O-H was at 1638 cm^-1^ caused by crystal water. The stretching vibrations of the Al-O_6_ octahedron were identified as the absorption peaks at 750 cm^-1^ and 540 cm^-1^ [41]. The peak at 1096 cm^-1^ corresponded to the asymmetric vibration of the Si-O-Si bond [42]. In addition, the peaks at 952 cm^-1^ was assigned to the deformation vibration of Al-OH. The FTIR results showed that the position of the characteristic peaks of the MLDHs did not change after adsorption. However, the intensity of the band at 952 cm^-1^ increased after adsorption, which reflected that the Cl^−^ intercalcation at interlayers affected the deformation vibration of -OH [24, 43].

**Fig 8:**
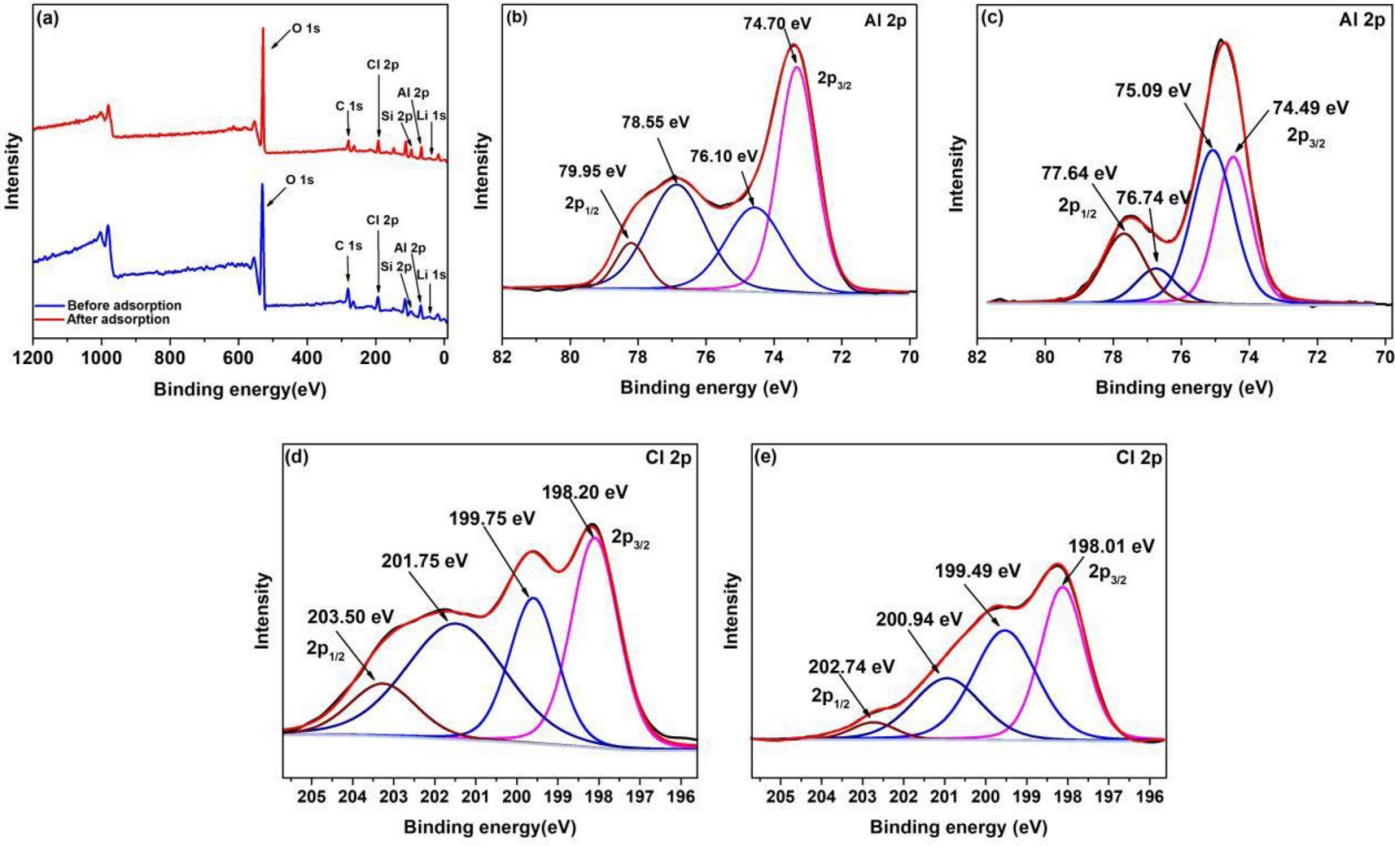
XPS spectra of MLDHs: (a) survey spectra, (b) Al2p spectra before adsorption, (c) Al2p spectra before adsorption, (d) Cl2p spectra before adsorption, (e) Cl2p spectra after adsorption.

The XPS spectra of the MLDHs before and after adsorption were exhibited in Fig 8 to unravel the element distribution and surface state of MLDHs. As shown in Fig 8A, the Li1s, O1s, C1s, Cl2p, Si2p and Al2p peaks were observed in wide scan of XPS spectra of MLDHs. However, the Fe2p peak was not observed in the wide scan of XPS spectra, which may be caused by the coating of Fe_3_O_4_ particles by SiO_2_. Before being used for lithium adsorption, only a fraction of Li^+^ need to be removed from the original structure of adsorbents. Therefore, the Li1s peak could be observed in the wide scan of XPS spectra of MLDHs before and after adsorption. Fig 8B and 8C showed the Al2p spectra resolutions of MLDHs before and after adsorption. The Al2p_3/2_ binding energy transformed from 74.70 eV and 76.10 eV to 74.49 eV and 75.09 eV, respectively, which indicated that the Li^+^ entered into the crystal structure of adsorbents and affected the chemical environment around the Al-O in the adsorbents [24]. It can be found from Fig 8D and 8e that the binding energy of Cl2p also changed after adsorption, which indicated that the Cl^-^ entered into the structure of adsorbents in order to maintain the charge balance.

#### 3.3.1 Adsorption kinetics

**Fig 9:**
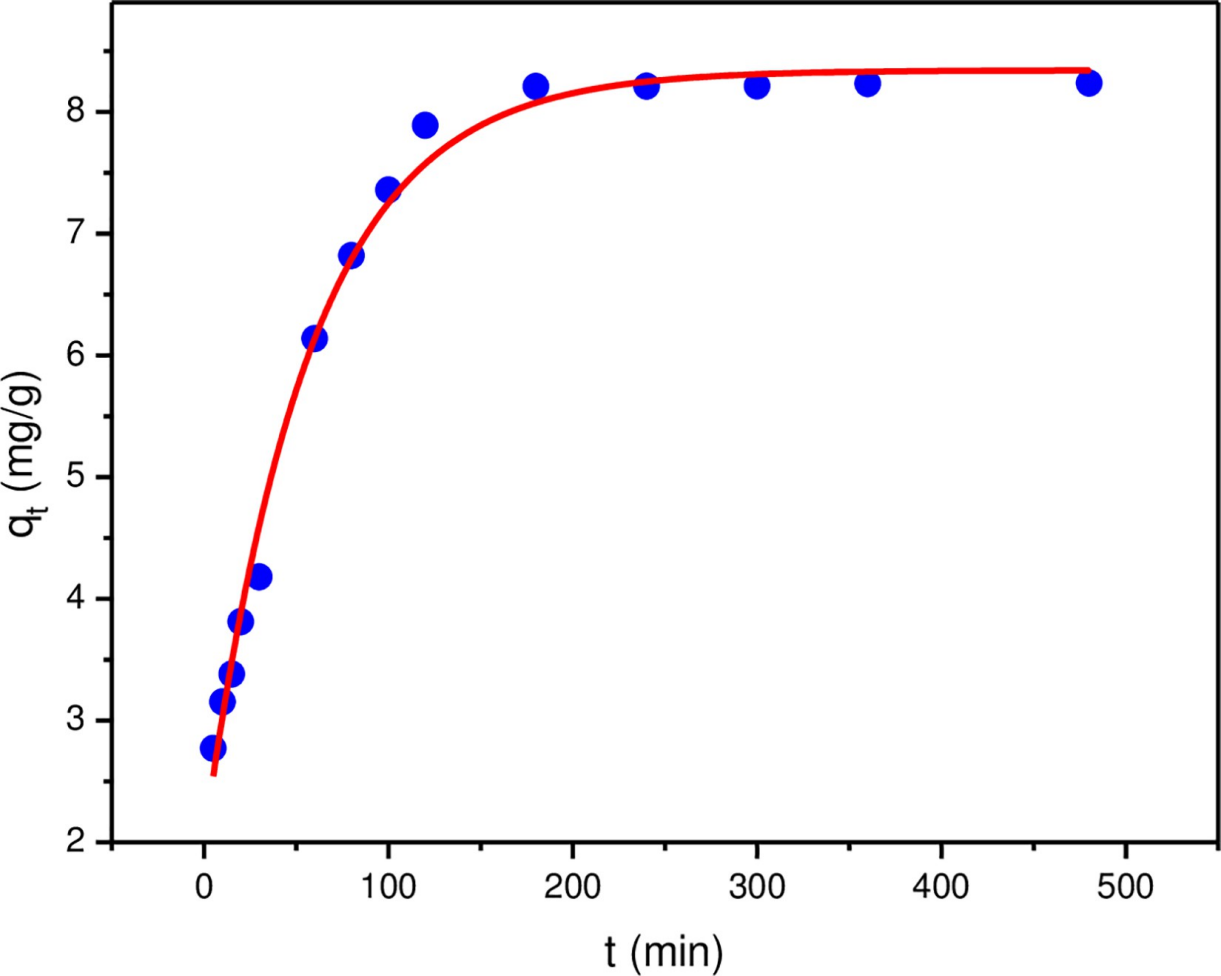
Effect of contact time on adsorption capacity.

The variation curve of adsorption capacity with contact time was shown in Fig 9. Obviously, the adsorption capacity of lithium ion changed rapidly with time. Specifically, The first 120 min corresponded to a rapid adsorption stage, which was mainly due to the sufficient amount of lithium ions that can be adsorbed and the adsorption materials can provide enough binding sites when the adsorbents reacted with lithium ions in the initial stage. As the contact time prolongs, the increasing rate of lithium ions adsorption capacity slowed down and finally tended to equilibrium. This was due to the gradual decrease in the available binding sites on the adsorbent material with increasing contact time. In the follow-up experiment, the oscillation time of the lithium ion adsorption experiment was determined to be 180 min.

Two kinetic models were used to describe the lithium adsorption process. The pseudo-first-order model was a conventional method used to describe the adsorption of adsorbates from the liquid phase onto the solid phase, corresponding to the diffusion-controlled process [44, 45]. Ho and McKay had proposed the pseudo-second-order kinetic model which based on the assumption that the rate limiting step may be chemical sorption or chemisorption [46, 47]. The pseudo-first-order kinetic model can be represented by Eq (5), and Eq (6) described the pseudo-second-order kinetic model. It is helpful to further understand the mechanism of the adsorption process by fitting the adsorption kinetic data.

$$\ln\left(q_{e}-q_{t}\right)=lnq_{e}-k_{1}∙t$$ (5)

$$\frac{t}{q_{t}}=\frac{1}{k_{2}q_{e}^{2}}+\frac{1}{q_{e}}t$$ (6)

where q_e_ and q_t_ represent the lithium adsorption capacity at equilibrium and time t, respectively. The K_2_ (g/(mg·min)) and K_1_ (min^-1^) are both adsorption rate constants.

**Fig 10:**
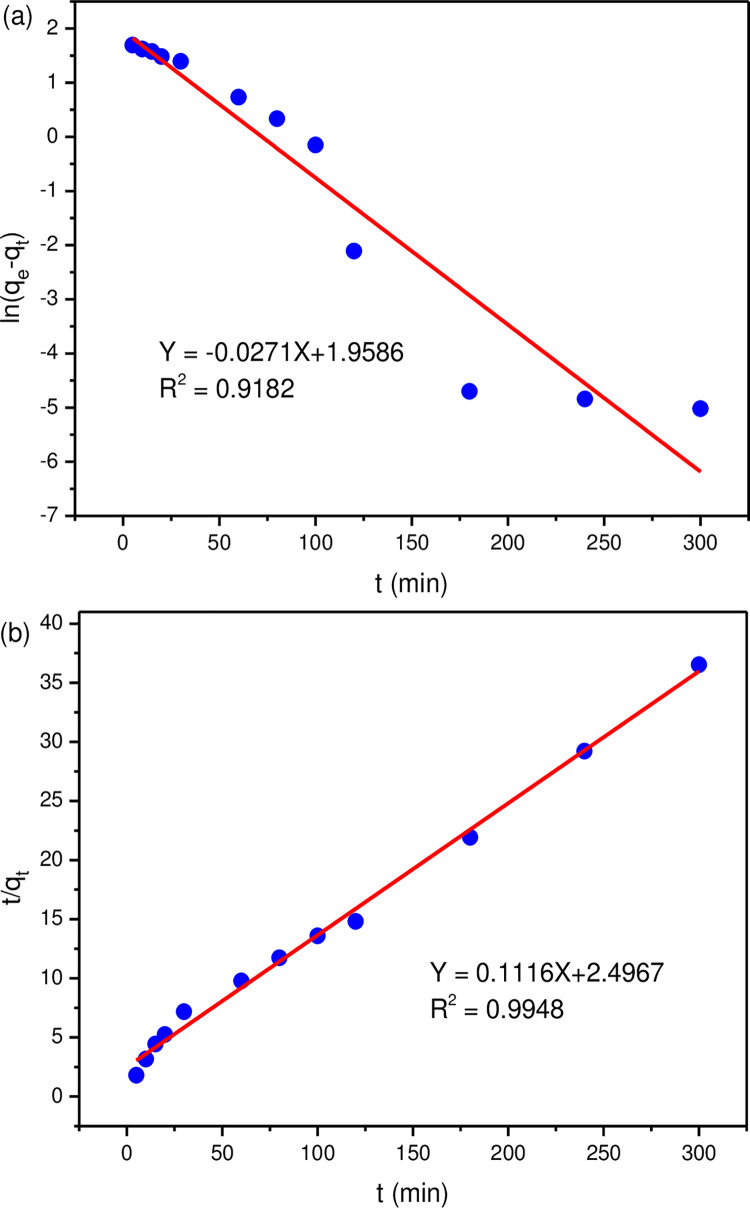
The fitting sorption data of MLDHs: (a) pseudo-first-order equation; (b) pseudo-second-order equation.

According to Eq (5) and Eq (6), the kinetic parameters of adsorption can be obtained by examining the linear relationship between ln (q_e_-q_t_) versus t and t/q_t_ versus t (Fig 10) and calculating their slope and intercept. Comparing the fitted correlation parameters R^2^ of the two kinetic models from the data in Table 1, it could be seen that the correlation parameter of pseudo-second-order kinetic model fitting reached 0.9948, while the correlation parameter of pseudo-first-order kinetic model fitting was only 0.9182. Therefore, the sorption kinetics of lithium were more appropriately described by the pseudo-second-order model. It can be inferred that the adsorption of lithium by the MLDHs may be a chemisorption-dominated process rather than a simple physical diffusion process.

**Table 1:** The fitting kinetic parameters.

kinetic model	*q*_e, exp_(mg g^-1^)	*q*_e, cal_(mg g^-1^)	*k*_*1*_(min^-1^)	*k*_*2*_(min mg g^-1^)	*R* ^*2*^
pseudo-first order	8.22	7.09	0.0271	/	0.9182
pseudo-second order	8.22	8.96	/	0.0050	0.9948

#### 3.3.2 Adsorption isotherms

**Fig 11:**
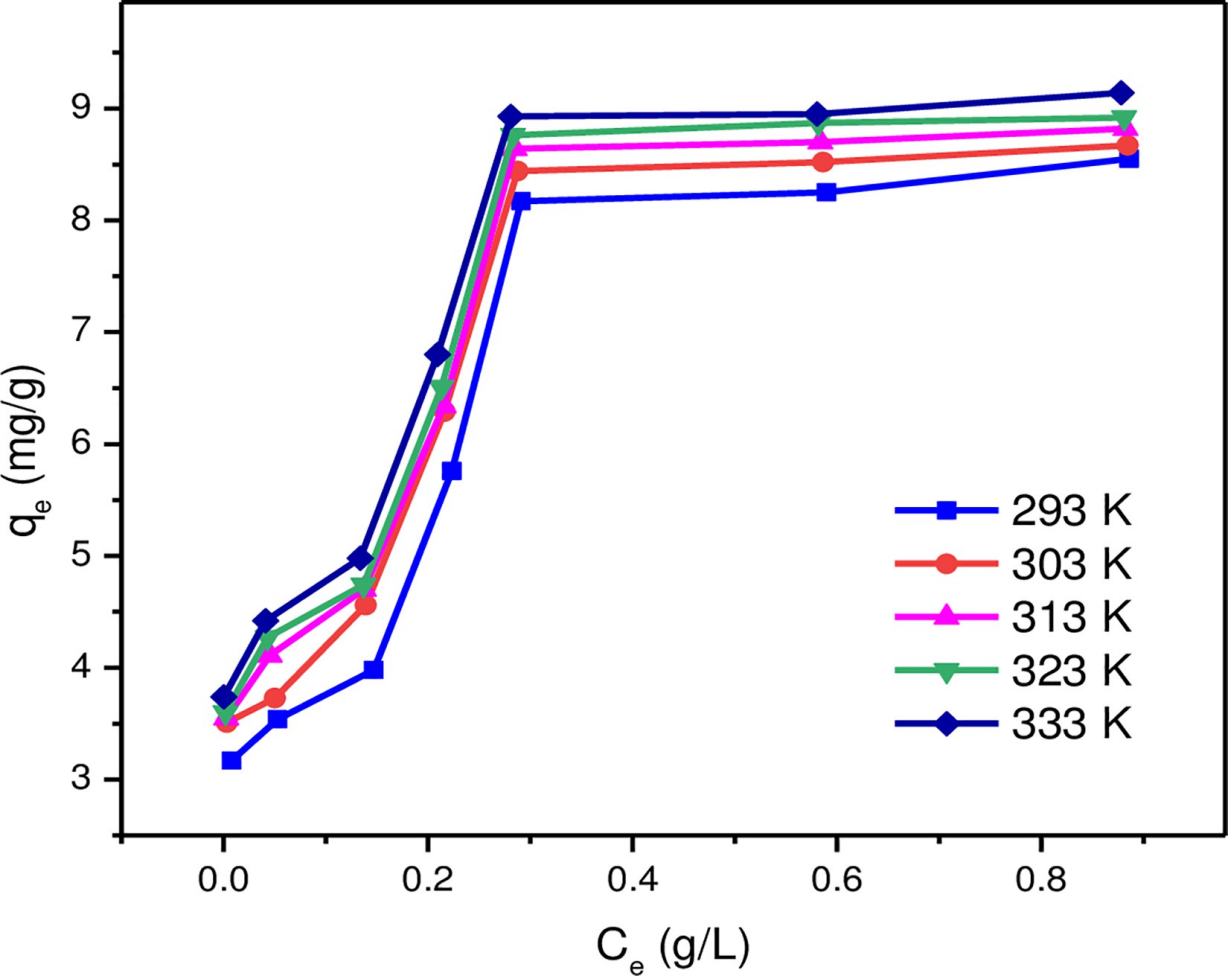
The Li^+^ adsorption isotherms of MLDHs under different temperatures.

In Fig 11, the adsorption isotherms of MLDHs measured at 293, 303, 313, 323 and 333 K revealed that the adsorption capacity of MLDHs had a positive correlation with the temperature and mildly increased with increasing temperature. Therefore, the adsorption process of lithium ions by MLDHs was endothermic.

The adsorption process of solid adsorbent in solution was usually described by the Langmuir and Freundlich adsorption isotherm models. The Langmuir isothermal adsorption model was an ideal single-molecule layer adsorption model with an expression as in Eq (7).

$$\frac{c_{e}}{q_{e}}=\frac{1}{bq_{m}}+\frac{c_{e}}{q_{m}}$$ (7)

where q_m_ (mg/g) and b (L/mg) are saturated adsorption capacity and Langmuir constant, respectively. The q_e_ (mg/g) and c_e_ (mg/L) are the lithium adsorption capacity and lithium concentration at equilibrium, respectively.

The Freundlich isotherm adsorption model was often used to describe the adsorption behavior on irregular surfaces and was expressed in Eq (8).


$$lnq_{e}=lnK_{F}+\frac{1}{n}lnc_{e}$$ (8)

Where K_F_ was the Freundlich model constant.

**Fig 12:**
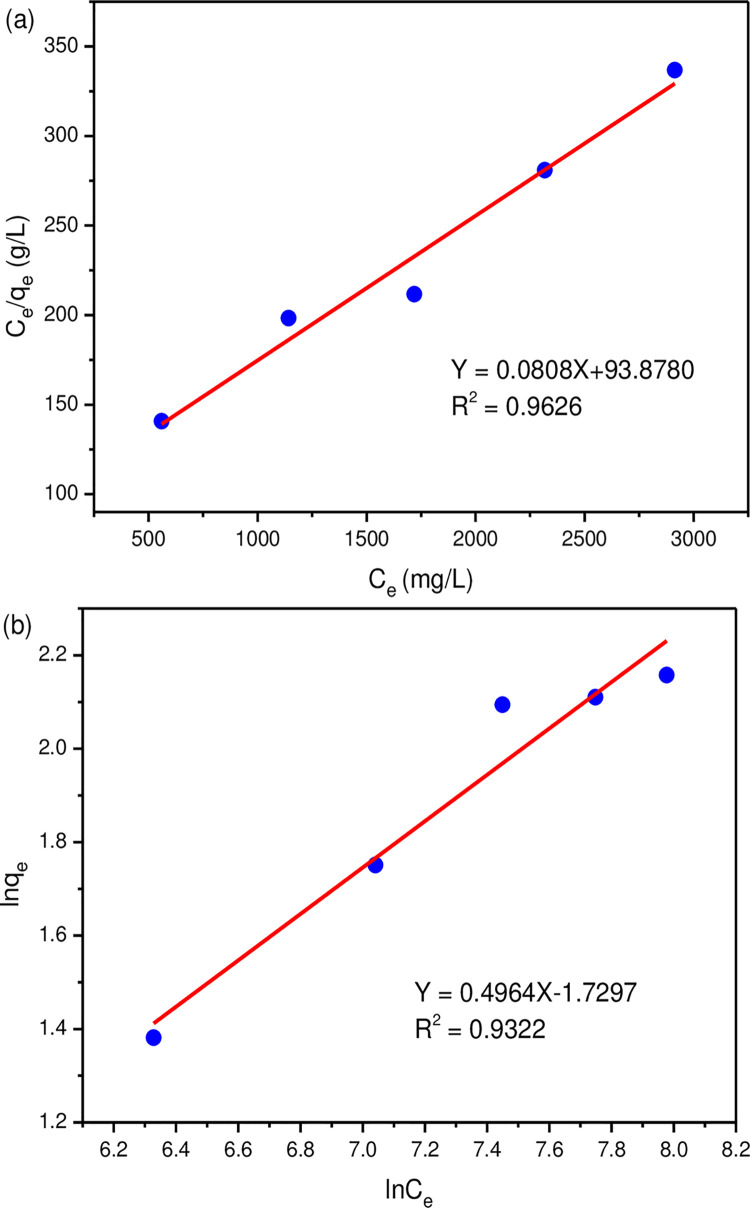
Isothermal adsorption model, (a) langmuir model, (b) freundlich model.

Fig 12 showed the adsorption isotherms of MLDHs, and the fitted parameters were listed in Table 2. The fitting coefficient of the Langmuir model was 0.9626, which was higher than that of the Freundlich model. The Langmuir model may be more suitable for explaining the adsorption of lithium ions. Therefore, the adsorption of lithium ions by MLDHs behaved as monolayer adsorption, which was a chemical adsorption process.

**Table 2:** Fitting parameters of the isothermal adsorption model (293 K).

Langmuir isotherm model			Freundlich isotherm model		
q_m_ (mg/g)	b (L/mg)	R^2^	n	K_F_	R^2^
12.38	0.0009	0.9626	2.0145	0.1773	0.9322

**Fig 13:**
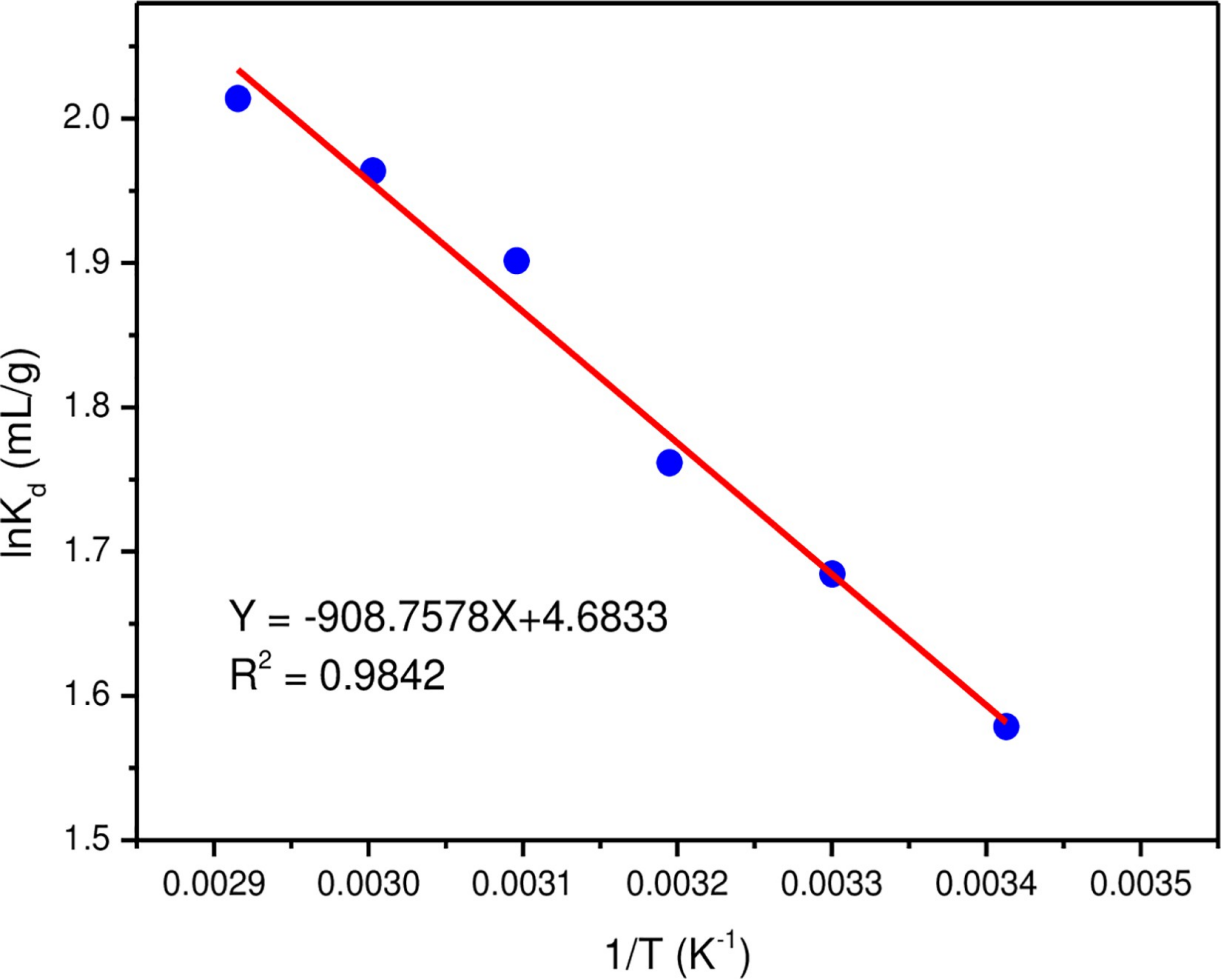
Plot of lnK_d_ versus 1/T.

The temperature usually affected on the adsorption capacity of MLDHs. The adsorption thermodynamic data of lithium ion were obtained by investigating the adsorption effect of MLDHs for lithium ion at different temperatures. According to Eq (9) and Eq (10), the thermodynamic parameters ΔH^0^, ΔS^0^ and ΔG^0^ of the adsorption system can be calculated. A plot of lnK_d_ versus 1/T yields a straight line. The slope of this line is -ΔH^0^/R and the intercept is ΔS^0^/R. From Fig 13, ΔH^0^ and ΔS^0^ values can be obtained and listed in Table 3. It can be seen from Table 3 that ΔH^0^ was positive, which indicated that the adsorption of lithium ions by MLDHs was endothermic, and the increase in temperature was beneficial to improve the adsorption capacity of lithium ions. The value of ΔS^0^ obtained was 38.94 J·mol^-1^·K^-1^, which indicated that the adsorption process of lithium ions was an entropy-increasing process. The adsorption temperature varied from 293 K to 343 K, and the obtained ΔG^0^ values were all negative. Therefore, the adsorption process of lithium ions by MLDHs was spontaneous under practical conditions. In addition, it can be found that the value of ΔG^0^ decreased continuously with increasing temperature. The increasing temperature was beneficial to increase the adsorption capacity of MLDHs.


$$\Delta G^{0}=-RT\ln K_{d}$$ (9)

$$\ln K_{d}=\frac{\Delta S^{0}}{R}-\frac{\Delta H^{0}}{RT}$$ (10)

**Table 3:** Adsorption thermodynamic parameters of MLDHs for lithium ion adsorption.

*T* (K)	*ΔG*^*0*^ (kJ/mol)	*ΔS*^*0*^ (J/(mol^.^K))	*ΔH*^*0*^ (kJ/mol)
293	-3.85	38.94	7.56
303	-4.24		
313	-4.58		
323	-5.11		
333	-5.44		
343	-5.74		

### 3.4 Adsorption of lithium ions from multi-metal-ion solutions

**Fig 14:**
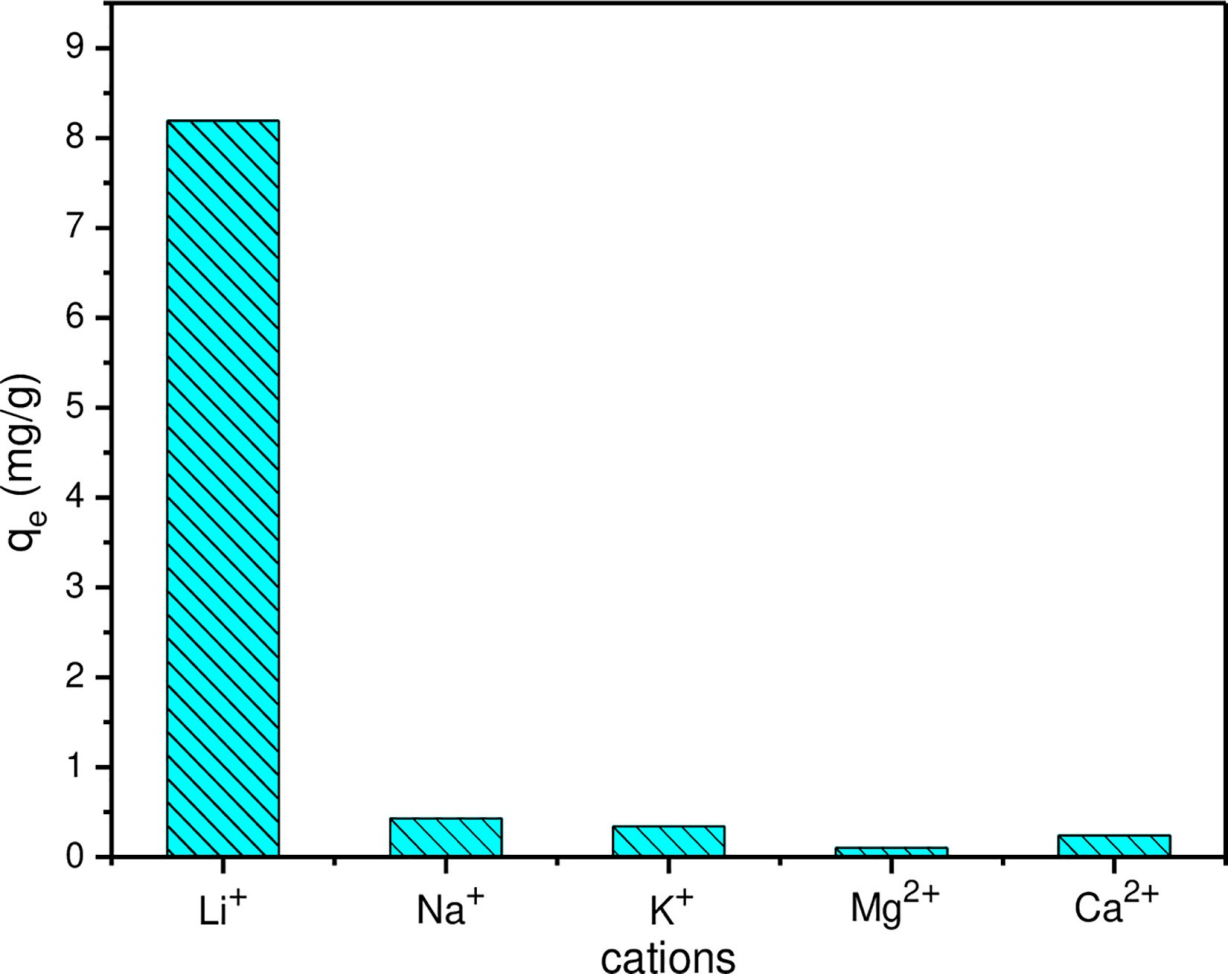
Adsorption amount of metal ion in solution by magnetic aluminum salt lithium adsorbent.

The selectivity of MLDHs for lithium ion in brine solution was investigated. The brine was prepared in the laboratory and its composition was referred to the old brine of the Qarhan Salt Lake in China. The ion concentrations in simulated brine were as follows: Li^+^, 0.412 g·L^−1^; Mg^2+^, 110.539 g·L^−1^; K^+^, 0.474 g·L^−1^; Na^+^, 1.316 g·L^−1^; Ca^2+^, 0.0518 g·L^−1^. In the experiment, 0.2 g MLDHs were added to 15 mL brine solution and shaken for 180 min. The adsorption capacity and separation factor $\alpha_{Me}^{Li}$ (Me = Na^+^, K^+^, Ca^2+^ and Mg^2+^) were calculated. As can be seen from Fig 14, the MLDHs have a small adsorption capacity for other metal ions in the mixed metal ion solution. The corresponding $\alpha_{Na}^{Li},\alpha_{K}^{Li},\alpha_{Ca}^{Li}$ and $\alpha_{Mg}^{Li}$ values were 23.52, 29.82, 42.34 and 101.97, respectively. The experimental results showed that the MLDHs had good selectivity for lithium ions. Due to the size-matched vacancies and ion memory effect of lithium ions, MLDHs exhibited high selectivity for lithium ions [48]. The adsorption capacity of several aluminum-based adsorbents was listed in Table 4. It can be found that the MLDHs in this work have a higher adsorption capacity compared to those reported in the literature.

**Table 4:** Comparison of Li/Al-LDHs adsorbents.

Adsorbents	q_e_ (mg/g)	References
Li/Al-LDHs	4.92	[32]
Li/Al-LDHs	7.27	[24]
Li/Al-LDHs	< 7	[49]
Li/Al-LDHs	5.69	[50]
MLDHs	5.83	[51]
MLDHs	8.22	This work

### 3.5 Adsorption-desorption cycles

**Fig 15:**
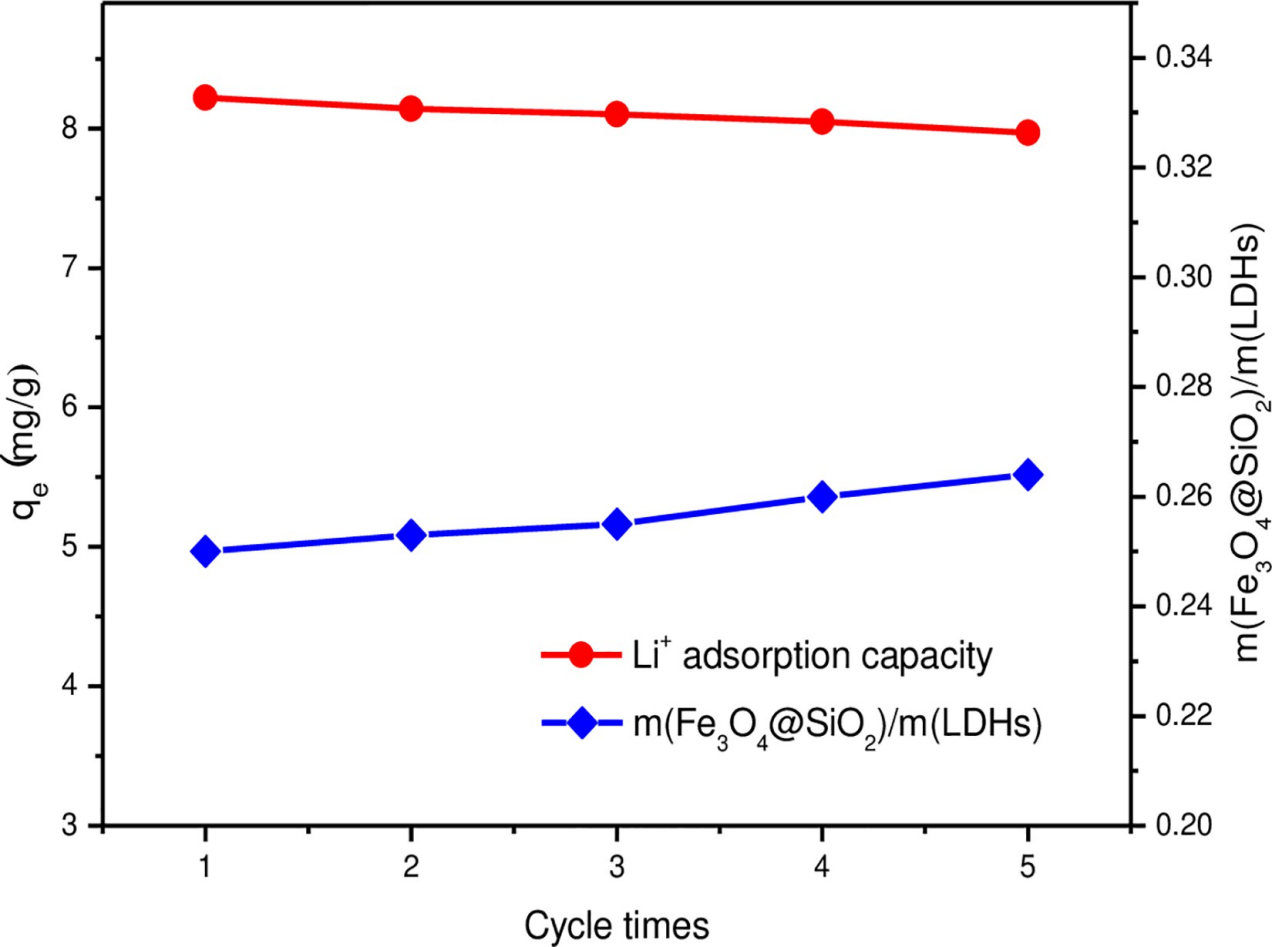
Li^+^ adsorption capacities and mass ratio of Fe_3_O_4_@SiO_2_ magnetic particles to LDHs changing with the number of adsorption-desorption cycles.

The adsorption properties of Li^+^ in solution by five adsorption-desorption cycles were studied to evaluate the stability and adsorption performance of MLDHs. The Li-loaded MLDHs were quickly washed with ice water (The ratio of the mass of MLDHs to the volume of water was 1 g/2 mL) to remove the residual brine remaining on the surface of the adsorbents, and dried at 60°C for 10 h. Then the Li^+^ in MLDHs was desorbed with deionized water at room temperature (The ratio of the mass of MLDHs to the volume of water was 1 g/50 mL), and shaken at 120 rpm for 6 h. The desorbed MLDHs were separated from the aqueous solution, dried at 60°C for 10 h, and used for the next adsorption cycle. The changing trend of Li^+^ adsorption capacities and mass ratio of Fe_3_O_4_@SiO_2_ magnetic particles to LDHs in MLDHs were shown in Fig 15. The mass ratio of Fe_3_O_4_@SiO_2_ magnetic particles to LDHs in MLDHs was 1:4 before adsorption. The MLDHs (0.2 g) was used in the adsorption-desorption cycle experiments, and the MLDHs solid was separated from the solution by a permanent magnet. The supernatant was collected and the unrecovered MLDHs particles were all dissolved by adding nitric acid. As control, 0.20 g MLDHs in 15 mL suspension was also dissolved by nitric acid. The Al^3+^ and Fe^3+^ concentrations in the solution were determined by ICP-OES, and the masses of Fe_3_O_4_@SiO_2_ magnetic particles and LDHs lost to the solution in each adsorption-desorption cycle experiment were calculated. It was found from Fig 15 that the mass ratio of Fe_3_O_4_@SiO_2_ magnetic particles to LDHs increased slightly with the increase of the number of adsorption-desorption cycles. Therefore, a small amount of LDHs was lost to the solution during the adsorption-desorption experiments. Since the LDHs were effective adsorption components in MLDHs, the adsorption capacity of MLDHs for Li^+^ also decreased slightly with the increase in the number of adsorption-desorption cycles. After five adsorption-desorption cycles, the adsorption capacity of magnetic aluminum-based lithium adsorbent decreased from 8.22 mg/g to 7.97mg/g, which maintained 96.96% of the initial adsorption capacity.

**Fig 16:**
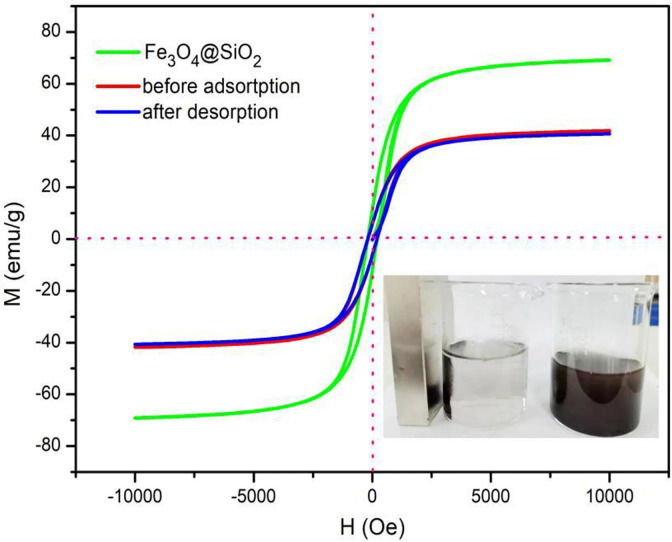
The magnetic hysteresis loops of Fe_3_O_4_@SiO_2_ and MLDHs.

The magnetic hysteresis loops of MLDHs before and after adsorption were further measured and compared with those of Fe_3_O_4_@SiO_2_. Obviously, these hysteresis loops all present typical S-shaped curves in Fig 16. Since Fe_3_O_4_@SiO_2_ nanoparticles were dispersed in the interlayer and surface of the aluminum-based adsorbents (LDHs), their saturation magnetizations were lower than that of pure Fe_3_O_4_@SiO_2_. However, when the magnet was close to the aqueous solution, the MLDHs will rapidly gather towards the magnet, which made the MLDHs separate from the aqueous solution. In addition, it can be observed that the magnetic hysteresis loops of MLDHs coincide before and after adsorption, which indicated that MLDHs had good stability and less solution loss in the process of adsorption.

## 4. Conclusion

In this study, MLDHs were prepared and applied for the separation of lithium ions from aqueous solutions. The adsorption capacity of MLDHs for lithium ions reached 8.22 mg/g in the static adsorption experiment. In the mixed solution of various metal ions, the corresponding $\alpha_{Na}^{Li},\alpha_{K}^{Li},\alpha_{Ca}^{Li}$ and $\alpha_{Mg}^{Li}$ values were 23.52, 29.82, 42.34 and 101.97, respectively. The experimental results show that the MLDHs have good selectivity for lithium ions. Kinetic studies indicated that the adsorption process conformed to a pseudo-second-order model. The Langmuir model may be more suitable for explaining the adsorption of lithium ions. The thermodynamic parameters revealed that the adsorption of lithium was a spontaneous endothermic process. In addition, the results of five adsorption-desorption cycles showed that MLDHs exhibited good stability and adsorption performance.
